# Electrochemical degradation of surfactants in domestic wastewater using a DiaClean^®^ cell equipped with a boron-doped diamond electrode

**DOI:** 10.3389/fchem.2023.900670

**Published:** 2023-04-25

**Authors:** Dayana G. Cisneros-León, Patricio J. Espinoza-Montero, Diego Bolaños-Mendez, Jocelyne Alvarez-Paguay, Lenys Fernández, Pablo F. Saavedra-Alulema, Kelly Lopez, Diana Astorga, José Luis Piñeiros

**Affiliations:** ^1^ Escuela de Ciencia Químicas, Pontificia Universidad Católica del Ecuador, Quito, Ecuador; ^2^ Departamento de Ingeniería Civil y Ambiental, Escuela Politécnica Nacional, Quito, Ecuador; ^3^ Facultad de Ciencias Químicas, Universidad Central del Ecuador, Quito, Ecuador; ^4^ Escuela de Ciencias Biológicas, Pontificia Universidad Católica del Ecuador, Quito, Ecuador

**Keywords:** municipal wastewater, surfactants degradation, electrocatalysis, electrochemical water treatment, electrochemical oxidation, advanced oxidation processes, boron-doped diamond, reactive oxygen species

## Abstract

Treating domestic wastewater has become more and more complicated due to the high content of different types of detergents. In this context, advanced electro-oxidation (AEO) has become a powerful tool for complex wastewater remediation. The electrochemical degradation of surfactants present in domestic wastewater was carried out using a DiaClean^®^ cell in a recirculation system equipped with boron-doped diamond (BDD) as the anode and stainless steel as the cathode. The effect of recirculation flow (1.5, 4.0 and 7.0 L min^−1^) and the applied current density (j = 7, 14, 20, 30, 40, and 50 mA cm^−2^) was studied. The degradation was followed by the concentration of surfactants, chemical oxygen demand (COD), and turbidity. pH value, conductivity, temperature, sulfates, nitrates, phosphates, and chlorides were also evaluated. Toxicity assays were studied through evaluating *Chlorella sp*. performance at 0, 3, and 7 h of treatment. Finally, the mineralization was followed by total organic carbon (TOC) under optimal operating conditions. The results showed that applying j = 14 mA cm^−2^ and a flow rate of 1.5 L min^−1^ during 7 h of electrolysis were the best conditions for the efficient mineralization of wastewater, achieving the removal of 64.7% of surfactants, 48.7% of COD, 24.9% of turbidity, and 44.9% of mineralization analyzed by the removal of TOC. The toxicity assays showed that *Chlorella* microalgae were unable to grow in AEO-treated wastewater (cellular density: 0 × 10^4^ cells ml^−1^ after 3- and 7-h treatments). Finally, the energy consumption was analyzed, and the operating cost of 1.40 USD m^−3^ was calculated. Therefore, this technology allows for the degradation of complex and stable molecules such as surfactants in real and complex wastewater, if toxicity is not taken into account.

## 1 Introduction

The presence of persistent organic pollutants (POPs) in water, such as surfactants, poses adverse threats to ecosystems and human health. Surfactants are present in the formulation of detergents and other cleaning products, such as, disinfectants, cosmetics, and other personal care products (Armijos-Alcocer et al., 2017; Martínez Peña, 2017). These are amphiphilic molecules consisting of a polar (water-soluble) group and a non-polar group. Depending on the charge of their polar group, they are classified as anionic, cationic, non-ionic, and amphoteric (Ivanković & Hrenović, 2010; Palmer & Hatley, 2018, Figure 1).

**FIGURE 1:**
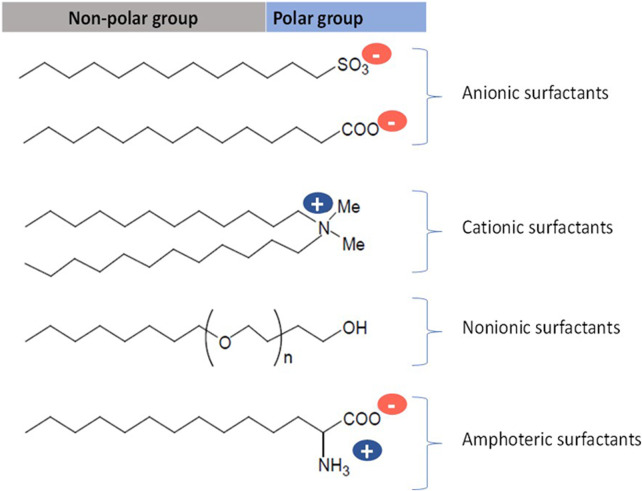
Classification of surfactants.

The concentration of surfactants increases permanently in the environment, especially in aqueous matrices, due to the increase in wastewater discharges from domestic and industrial cleaning activities without previous treatment (Salazar, 2014). High levels of surfactants severely affect aquatic environments due to their high toxicity in various organisms such as bacteria, plants, invertebrates, and aquatic vertebrates (Ivanković & Hrenović, 2010). Furthermore, a high concentration of surfactants causes high values in the chemical oxygen demand due to their structure, which stands as evidence for the level of pollution in water (Pillai et al., 2018). Although some treatment processes have been applied for realistic wastewater remediation, the results have not been entirely satisfactory due to the formation of highly toxic and even more persistent by-products. For example, absorption and biodegradation processes have been reported as conventional domestic wastewater treatments with high surfactant concentration (Cajas Oña, 2018; Ochoa-Chavez et al., 2018; Jardak et al., 2016). In biological processes, under aerobic and anaerobic conditions, the degradation pathway involves the formation of sulfophenyl carboxylic acids which are the major degradation intermediates and have been observed in wastewaters and in the environment (Eichhorn & Braunschweig, 2001). Moreover, benzenesulfonic acid, benzaldehyde, toluene, and benzene have been suggested as intermediates of degradation under anaerobic conditions (Carosia et al., 2014; Mogensen & Ahring, 2002). Many of these compounds increase the risk of carcinogenicity, mutagenicity, and endocrine disruption in aquatic life (Eichhorn & Braunschweig, 2001). On the contrary, the advanced oxidation processes (AOPs) appear as the simplest and most effective alternative to degrading highly stable compounds from wastewater. These processes are able to mineralize the organic matter present in water, which means to transform it into CO_2_, H_2_O, and inorganic ions (Armijos, 2016; Brillas et al., 2009; Espinoza-Montero et al., 2013; Espinoza-Montero et al., 2020). Nevertheless, an ecotoxicological assay is always recommended to ensure water quality at the end of treatment.

Advanced electrochemical oxidation (AEO) using boron-doped diamond (BDD) as an anode has been a prominent method for degrading POPs from water to complete mineralization. AEO consists of the electrogeneration of highly oxidizing reactive species (HORS). AEO involves direct and indirect oxidation simultaneously. In direct AEO, the oxidation of organic matter (charge transfer) occurs directly on the electrode surface; indirect AEO occurs through electrogenerated HORS on the electrode surface. These species can be radical (e.g., hydroxyl radicals (^•^OH), sulfate radical ( ${SO}_{4}^{•-}$ ), carbonate radical ( ${CO}_{3}^{•-}$ ), superoxide ion ( $O_{2}^{•-}$ ), singlet oxygen (^1^O_2_)) or non-radical (e.g., chlorine species chlorine (Cl_2_), hypochlorous acid (HClO), hypochlorous ion (ClO^−^), and chlorine dioxide (ClO_2_); peroxydisulfate ( ${S_{2}O}_{8}^{2-}$ ) and peroxydicarbonate $\left({C_{2}O}_{6}^{2-}\right.$ ) anions and hydrogen peroxide (H_2_O_2_)) (Espinoza-Montero et al., 2022; Soliu Oladejo Ganiyu et al., 2021). In real wastewaters, we can find a great variety of inorganic ions (e.g., Cl^−^, HCO_3_^−^/CO_3_^2-^, SO_4_^2-^ and dissolved oxygen) that favors the electrogeneration of HORS, which are able to oxidize organic matter present in the aqueous medium until its complete mineralization (reactions 1, 2, and 3) (Armijos-Alcocer et al., 2017; Moreira et al., 2017; De Battisti & Martínez-Huitle, 2018; Espinoza-Montero et al., 2020; Carrera-Cevallos et al., 2021; He et al., 2022). On BDD, the direct electro-oxidation (EO) of organic compounds (R) occurs according to reaction (1), and the indirect/mediated oxidation occurs via HORS, electrogenerated on the anode, such as the physisorbed hydroxyl radical BDD (^•^OH) (reactions (2) and (3)) and other HORS species as given in the following reactions:

$$BDD+R_{red}\;\to \;BDD+{ze}^{-}+R_{ox}$$ (1)

$$BDD+H_{2}O\;⇄\;BDD\left[\left.{(}^{•}OH\right)\right]+H^{+}+e^{‒}$$ (2)

$$R+BDD{(}^{•}OH)\to BDD+{xCO}_{2}+{yH}_{2}O+z\left(inorganic\;ions\right)$$ (3)

Using BDD by electrochemical oxidation (EO) in the $HCO_{3}^{-}$ / $HCO_{3}^{2-}$ medium, $HCO_{3}^{•-}$ y $C_{2}O_{6}^{2-}$ are produced by direct and indirect oxidation from $HCO_{3}^{-}$ / $HCO_{3}^{2-}$ (reactions 4–8) by e^−^ and BDD (^•^OH) transfer (Ruiz et al., 2014; Ganiyu & El-din, 2020). In addition, reactive sulfate species ( ${S_{2}O}_{8}^{2-}$ and ${SO}_{4}^{•-}$ ) are generated on BDD by EO in the presence of sulfate ( ${SO}_{4}^{2-}$ ). ${S_{2}O}_{8}^{2-}$ is generated through: 1) oxidation of ${SO}_{4}^{2-}$ at the anode, 2) oxidation of ${SO}_{4}^{2-}$ with electrogenerated ^•^OH and 3) recombination of produced ${SO}_{4}^{•-}$ (reactions 9, 10 and 11, respectively). On the other hand, ${SO}_{4}^{•-}$ can be generated by: 1) oxidation of ${SO}_{4}^{2-}$ at the anode (reaction 12) and 2) oxidation of ${SO}_{4}^{2-}$ by electrogenerated ^•^OH (reaction 13) (Cai et al., 2019; Soliu O Ganiyu & El-din, 2020).

$$CO_{3}^{2-}\to CO_{3}^{•-}+e^{-}$$ (4)

$$BDD\left({\;}^{•}OH\right)+CO_{3}^{2-}\to BDD+CO_{3}^{•-}+{OH}^{-}$$ (5)

$$2HCO_{3}^{-}+2BDD\left({\;}^{•}OH\right)\to 2BDD+{C_{2}O}_{6}^{2-}+2H_{2}O$$ (6)

$$2CO_{3}^{2-}\to {C_{2}O}_{6}^{2-}+2e^{-}$$ (7)

$$2CO_{3}^{•-}\to {C_{2}O}_{6}^{2-}$$ (8)

$$2{SO}_{4}^{2-}\to S_{2}O_{8}^{2-}+2e^{-}$$ (9)

$$12H{SO}_{4}^{-}+2^{•}OH\to S_{2}O_{8}^{2-}+2H_{2}O$$ (10)

$${SO}_{4}^{•-}+{SO}_{4}^{•-}\to S_{2}O_{8}^{2-}$$ (11)

$${SO}_{4}^{-}+e^{-}\to {SO}_{4}^{•-}$$ (12)

$${SO}_{4}^{2-}+{\;}^{•}OH\to {SO}_{4}^{•-}+H^{+}$$ (13)

In addition, on BDD, Cl^•^ (reaction 17) and $ClO_{2}^{•}$ (reaction 18) can be generated by EO in the chloride medium (Jasper et al., 2017). Depending on pH conditions and Cl^−^ concentration in the effluent, Cl_2_ is initially produced by oxidation of Cl^−^ to Cl_2_ (reaction 14), which then reacts with H_2_O to generate the weak acid HOCl (reaction 15), which is in equilibrium with ClO^−^ depending on the pH of the solution (reaction 16) (Soliu O. Ganiyu & Martínez-Huitle, 2019).

$$2{Cl}^{-}\to {Cl}_{2}+2e^{-}$$ (14)

$${Cl}_{2}+H_{2}O\to HOCl+H^{+}+{Cl}^{-}$$ (15)

$$HOCl⇄{ClO}^{-}+H^{+}$$ (16)

$${Cl}^{-}\to {Cl}^{•}+e^{-}$$ (17)

$$ClO_{2}^{-}\to ClO_{2}^{•}+e^{-}$$ (18)

Finally, on BDD, peroxodiphosphate ( ${P_{2}O}_{8}^{4-}$ ) can be generated by EO in phosphate media (reaction 19). ${P_{2}O}_{8}^{4-}$ is a HORS that after forming can act immediately to degrade organic matter (Cañizares et al., 2005; Sánchez et al., 2013).

$$\begin{array}{c} {2PO}_{4}^{3-}\overset{BDD}{\to }{P_{2}O}_{8}^{4-}+{2e}^{-}\\ E{}^{\circ}=2.07\;V\;vs.\;NHE \end{array}$$ (19)

Furthermore, it has been reported that AEO has interesting features, such as compatibility, cost-effectiveness, easy implementation, and high efficiency (Einaga, 2014; Barrera-Díaz et al., 2018; Song et al., 2018). Nevertheless, the nature of anode materials plays an important role in the treatment efficiency, degradation mechanism, and selectivity (Espinoza et al., 2021; He et al., 2022). The BDD has demonstrated high efficacy compared to other types of electrodes owing to its wide electrochemical potential window, good chemical/electrochemical stability, and strong corrosion resistance (Armijos-Alcocer et al., 2017; Espinoza-Montero et al., 2013, 2022Rabbani et al., 2017) because of which this diamond electrode has been recognized as the most efficient and ideal anode material, which has been extensively investigated for the degradation of organic contaminants in aquatic environments (C. Dartsch & Pupunat, 2017; Carrera-Cevallos et al., 2021; Espinoza-Montero et al., 2022; He et al., 2022; Oturan & Oturan, 2018).

Even though surfactants have negative environmental effects, there is no further information on the treatment of domestic wastewater by AEO using BDD electrodes. Only few reports talked about the degradation of detergents by AEO, which show that surfactants are effectively removed from water (Armijos-Alcocer et al., 2017; Cajas Oña, 2018; Guanoluiza Llive, 2013; Rodríguez-Peña et al., 2019). However, previous reports have been carried out using synthetic wastewaters with a defined concentration of surfactants. On the other hand, it is important to highlight that BDD has been used successfully in the electrochemical treatment of leachate from sanitary landfills (Fernandes et al., 2013).

In this paper, AEO is proposed as a viable and efficient alternative for the treatment of domestic wastewater with a high concentration of surfactants. A commercial DiaClean^®^ cell equipped with a BDD anode was used, due to its excellent mass transfer, hydrodynamics, and superior current distribution (Cano et al., 2016; Armijos-Alcocer et al., 2017). The effect of current density and flow was studied in order to find the most favorable conditions for the degradation of domestic wastewater pollutants with high load of surfactants.

## 2 Experimental

### 2.1 Wastewater monitoring

The wastewater samples were taken from the effulent of the wastewater treatment plant in the “Arupos de la Hacienda” housing complex, located in Rumiñahui Canton in Ecuador. This plant treats wastewater generated by approximately 812 inhabitants (four inhabitants per household with a total of 203 homes for the housing complex). The parameters of wastewater samples were determined using the following procedures: the concentration of surfactants was determined by the HACH 8028 crystal violet method using a UV-VIS spectrophotometer HACH DR/4000 U (HACH COMPANY, 2000); mineralization was monitored through total organic carbon (TOC) following the Standard Methods 5310-C (2017) (Baird et al., 2018); and chemical oxygen demand (COD) was monitored according to USEPA HACH method 8000 (HACH COMPANY, 2000). Additionally, turbidity and pH values were monitored using a HACH DR/850 portable colorimeter and a pH meter (Mettler Toledo SevenCompact), respectively. Moreover, sulfates, nitrates, and phosphates were quantified using a HACH DR/4000U UV-Vis spectrophotometer. Chloride quantification was performed through the argentometric method from Standard Methods 4500-Cl B (Baird et al., 2018). Finally, conductivity and temperature were monitored using a Metrohm 912 conductivity meter and a digital multi-thermometer (−50°C to +300°C), respectively.

### 2.2 Wastewater degradation

**FIGURE 2:**
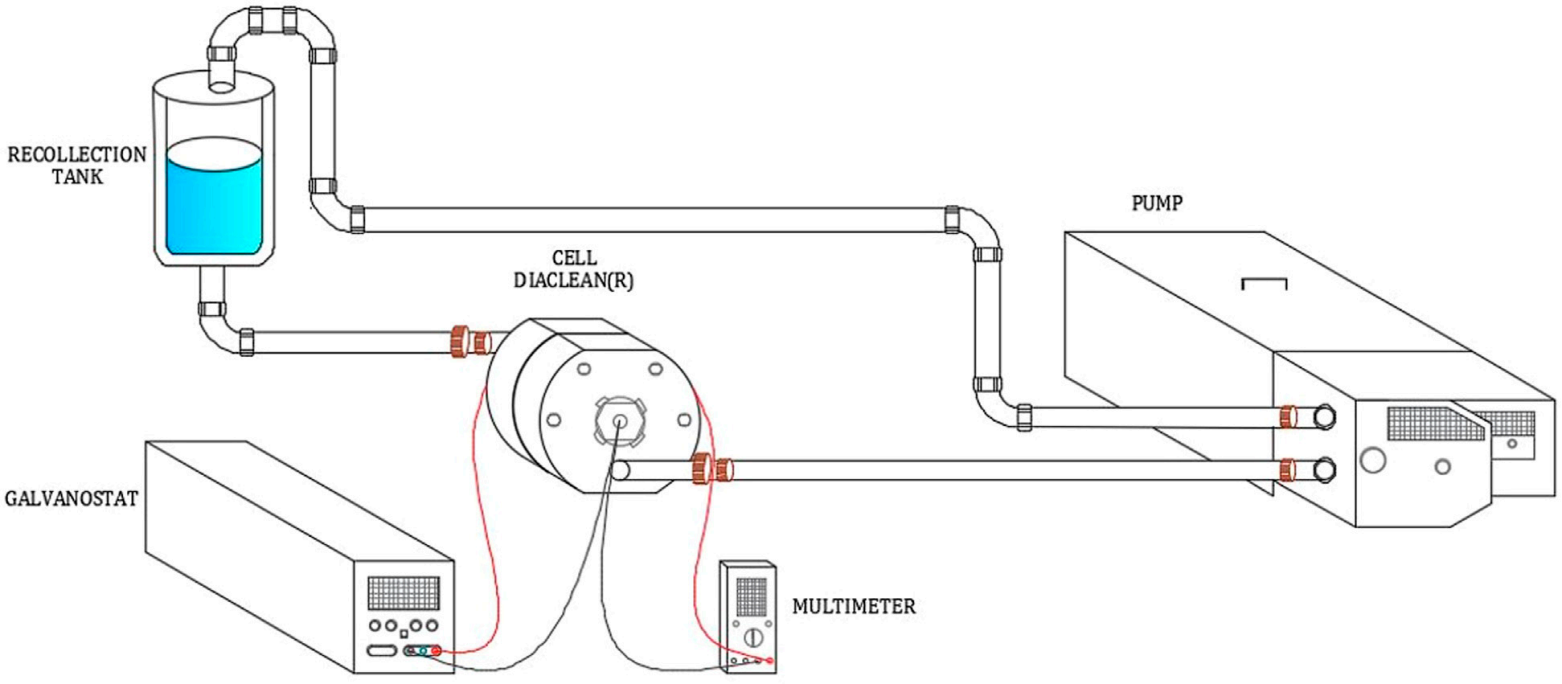
Experimental setup for the surfactant electrochemical degradation.

The degradation of wastewater samples was carried out under galvanostatic conditions coupled to a recirculation system (Figure 2). The DiaClean^®^ cell was coupled with a BDD disk electrode (Adamant Technologies, Switzerland) with a geometric circular surface of 70 cm^2^ (1–10 μm BDD thickness deposited on silicon with a resistance of 0.1 Ω cm) acting as an anode and a T304 stainless steel (SS) electrode with a geometric circular surface of 70 cm^2^ acting as an cathode. The cell was coupled to a peristaltic pump (MasterFlex I/P model 77600-62) to determine the best conditions among the flow rates (1.5, 4, and 7 L min^−1^), keeping the current density constant at 14.0 mA cm^−2^. Once the optimal flow rate was established, the effect of current density (7.0, 14.0, 20.0, 30.0, 40.0, and 50.0 mA cm^−2^) was studied using a GW INSTEK galvanostat, model SPS-3610, keeping the flow rate constant at 1.5 L min^−1^. This system was coupled to a multimeter (model 903-150N-B) for monitoring the applied current and determining the energy consumption of this system. The capacity of the recollection tank was 6 L. This volume was kept constant in all experiments, with each experiment carried out for 7 h. Moreover, prior to each test, the electrodes were cleaned with an acidic solution of H_2_SO_4_ (0.1 M), and a 2 A of current for 15 min was applied to remove impurities from their surface. Additionally, after 1 h, a sample was taken to monitor the efficiency of the AEO process by analyzing the concentration of surfactants, COD, TOC, turbidity, pH, conductivity, sulfates, nitrates, ferrum, phosphates, and chlorides, and temperature. Furthermore, to validate the reproducibility of the experimental procedure, each parameter was analyzed in triplicate.

The percentages of surfactant removal and mineralization in wastewater were determined using Eqs 1, 2, where S_o_ is the initial concentration of surfactants, S_f_ is the final concentration of surfactants, TOC_o_ is the total organic carbon, initial concentration, and TOC_f_ is the total organic carbon, final concentration.

$$Removal\;\left(\%\right)=\frac{\left(S_{o}-S_{t}\right)}{S_{o}}\times 100$$ (1)

$$Mineralization\;\left(\%\right)=\frac{\left({TOC}_{o}-{TOC}_{t}\right)}{{TOC}_{o}}\times 100$$ (2)

### 2.3 Ecotoxicity test with microalgae C*hlorella* sp.

The ecotoxicity test was carried out with a *Chlorella* strain provided by the Diserlab-PUCE Laboratory. The taxonomic confirmation and purity verification of the microalgal culture were accomplished with taxonomic keys and morphologic characteristics. The study was performed by means of aerated batch bioreactors using a 204.77 ± 53.216 × 10^4^ cell ml^−1^ algal inoculum and exposed to ambient temperature, with a 12/12-h photoperiod under direct solar light (Herrera et al., 2018), and one of the following treatment media was used per triplicate: Bristol (positive control), untreated wastewater (negative control), 3-h AEO-treated wastewater, and 7-h AEO-treated wastewater. The homogeneous distribution of nutrients and the adequate, similar, and constant flow of carbon dioxide were supplied by three-outlet aerators to guarantee optimal growth conditions. Algal cell density in Neubauer plates was evaluated per triplicate through cell counting under an optical microscope (40X) every 3 days for a 30-day period. For each treatment, the cell density was determined using Eq. 3 (Rodríguez et al., 2014).

$$DC=N\times 10^{4}\times Fd$$ (3)

where CD is the cellular density (×10^4^ cells ml^−1^), N is the average number of cells per 1 mm^2^ (0.1 µl), 10^4^ is the conversion factor (0.1 µl–1 ml), and Fd is the dilution factor.

### 2.4 Energy consumption and operational costs

The economic analysis of the electrochemical oxidation process takes into account the energy consumption of the DiaClean^®^ cell and peristaltic pump during 7 h of electrolysis. Energy consumption was estimated at 0.063 US·kW^−1^·h^−1^, which corresponds to its cost in educational institutions in Quito, Ecuador. In order to evaluate energy consumption, the equation proposed by Martínez Huitle and Brillas (2009) was applied to relate energy consumption to the treated sample volume in each run, as described in Eq. 4.

$$EC\;\left(kWhm^{-3}\right)=\frac{V∙A∙t}{1000∙Vs}$$ (4)

where V is the average potential (volts), A is the current applied in amps, t is the reaction time (hours), and Vs corresponds to the volume of the total sample treated (m^3^) (Ochoa-Chavez et al., 2018). Moreover, the energetic consumption of the peristaltic pump was also taken into account and determined according to Eq. 5, where P is the power of the peristaltic pump, which is 1 HP = 760 W, and t is the time of reaction.

EC Pump=P∙t (5)

Finally, the operational cost was determined by Eq. 6, where E is the total energy consumption, and the energy cost was estimated at 0.063 USD kW^−1^ h^−1^.

$$Cost=E∙\frac{cost}{1kWh}$$ (6)

## 3 Results and discussion

### 3.1 Effect of the flow rate on wastewater degradation

**FIGURE 3:**
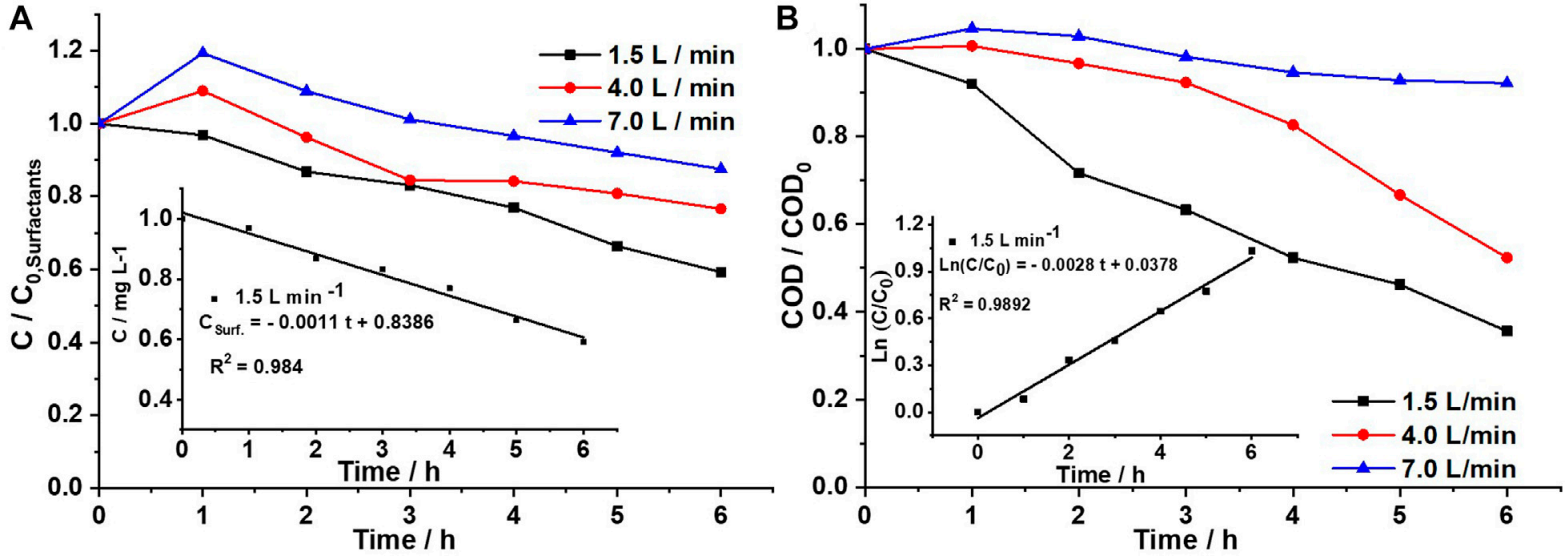
Effect of the recirculation flow rate on domestic wastewater electroremediation through **(A)** surfactant concentration and **(B)** COD, 14 mA cm^−2^, using a DiaClean^®^ cell coupled with BDD as an anode and SS as a cathode. Inset **(A)** Zero-order kinetics for surfactant degradation. Inset **(B)** Pseudo-first-order kinetics for COD removal.

Figure 3A shows the effect of flow on surfactant degradation at a constant current density (14 mA/cm^2^). At a lower flow rate (1.5 L min^−1^), the surfactant concentration decreases more effectively. Similarly, Figure 3B shows the COD removal at three different flow rates. These results indicate that the degradation process is affected by increasing the flow rate because residence time and mass transfer are limited inside the reactor. It means that at very high flow rates, there is less residence time for water inside the reactor, which reduces the interaction of domestic wastewater with the oxidizing species formed on the surface of the BDD electrode (Enciso et al., 2013). As a result, the optimal flow rate was determined to be 1.5 L min^−1^, reaching 64.7% removal of surfactants and 48.7% COD removal after 6 h of electrolysis. Moreover, the kinetic constant indicates that the degradation of a surfactant (*k* = 0.0011 min) (inset-Figure 3A) follows a zero-order kinetics and COD (*k =* 0.028 min^−1^) (inset-Figure 3B) follows a pseudo-first order reaction.

The results reported in this work were compared to those obtained in Guanoluiza et al. (2013). They conducted an electrochemical process in municipal wastewater purification and determined that at a highest flow rate there was good removal efficiency of contaminants (Guanoluiza Llive, 2013). In this work, it is important to highlight that at flow rates greater than 1.5 L min^−1^, large amounts of foam are generated due to the presence of detergents. In addition, the resistance of the samples increases considerably, causing heating of the cell. The presence of foam in bulk decreases the electrolytic conductivity and increases the electrical resistance.

### 3.2 Effect of current density on the domestic wastewater treatment

**FIGURE 4:**
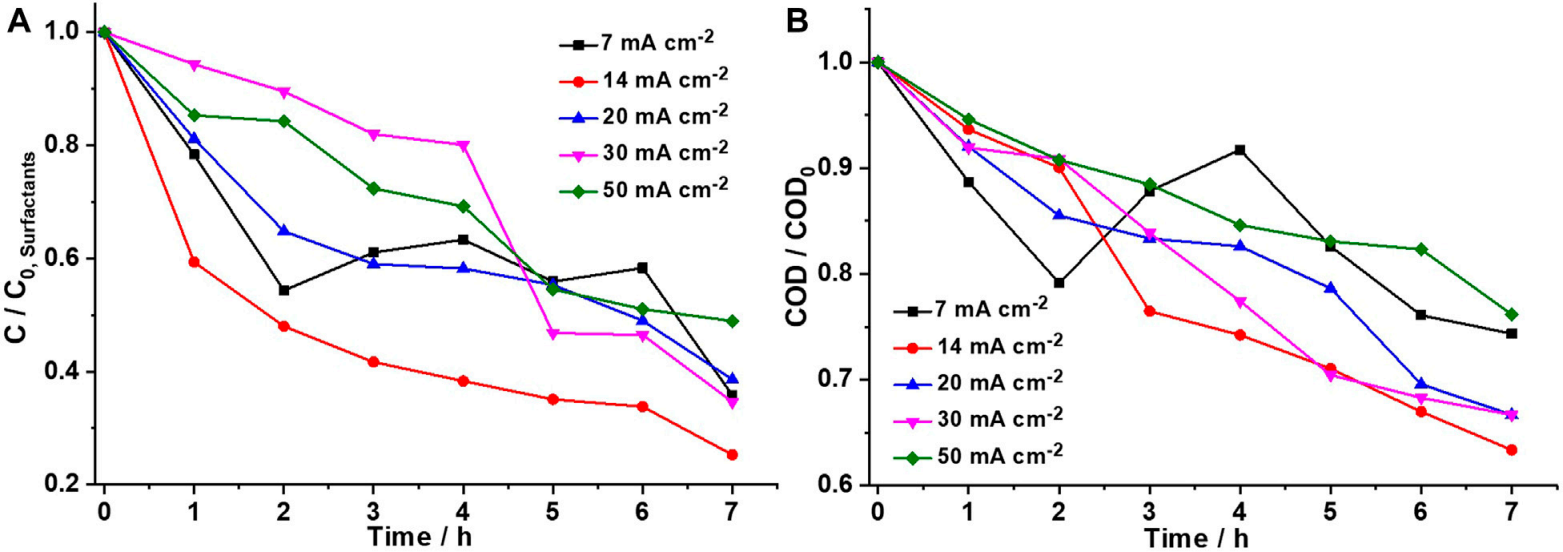
Effect of current density on domestic wastewater electroremediation through **(A)** surfactant concentration and **(B)** chemical organic demand at an optimal flow rate (1.5 L min^−1^) using a DiaClean^®^ cell coupled with BDD as an anode and SS as a cathode.

The effect of current density (7, 14, 20, 30, 40, and 50 mA cm^−2^) on the removal of surfactants and the mineralization of organic matter in domestic wastewater was studied, keeping the flow rate constant at 1.5 L min^−1^. The highest percentage of surfactant degradation (48.7%) and the highest COD removal (64.7%) were achieved at 14 mA cm^−2^ after 7 h of electrolysis (Figures 4A, B). In addition, as the current density increased, solution temperature and cell potential increased as well. However, a heat exchanger in the recollection tank helped maintain the constant initial temperature. In addition, the cell potential increases slightly due to foam formation and the consequent increase in medium resistance, which influences the energy consumption of the system. Finally, at current densities greater than 14 mA cm^−2^, the evolution of oxygen begins to favor the same as the generation of large amounts of foam. A similar behavior has been reported for the DiaClean^®^ cell equipped with BDD electrodes during the degradation of nonylphenol ethoxylate (NP_7_EO), a compound present in surfactants, humectants, dispersants, and emulsifiers (Armijos-Alcocer et al., 2017). Furthermore, Armijos-Alcocer et al. (2017) achieved the maximum rate of degradation of this surfactant at 40 mA cm^−2^. On the contrary, Carrera-Cevallos et al. (2021) reported that an increase in current density does not necessarily lead to a rise in the oxidation rate because the characteristics of the wastewater and mass transport limitations also affect the efficiency of the process. Nevertheless, the results reported in the aforementioned works were obtained using synthetic wastewater. Therefore, the present study is the first to use domestic wastewater from a small location in Ecuador.

### 3.3 Mineralization and final characterization of wastewater

**FIGURE 5:**
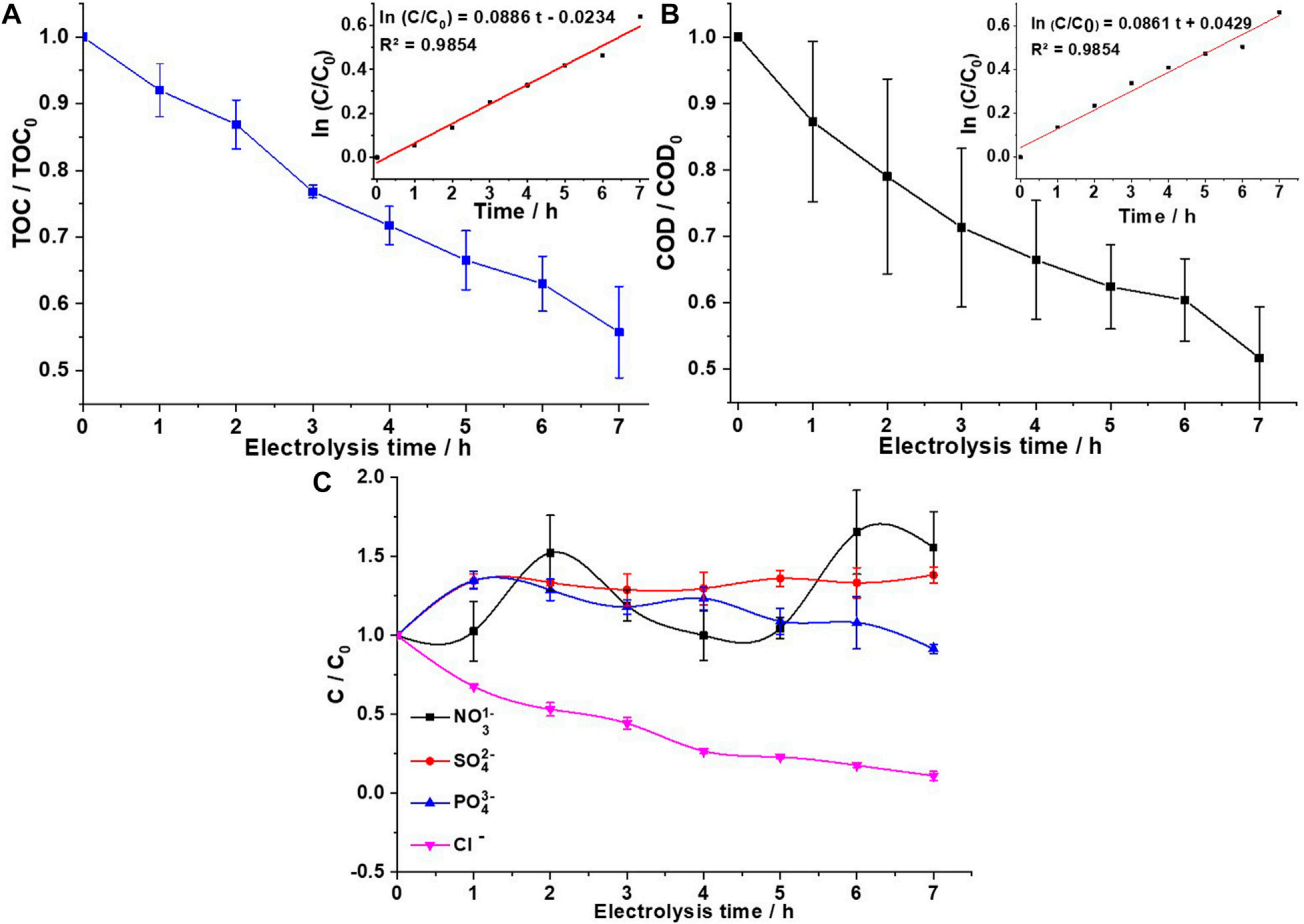
**(A)** TOC removal from domestic wastewater at a current density of 14 mA cm^−2^ and recirculation flow rate of 1.5 L min^−1^. Inset: Pseudo-first-order kinetics of TOC removal. **(B)** COD removal from domestic wastewater at a current density of 14 mA cm^−2^ and recirculation flow rate 1.5 L min^−1^. Inset: Pseudo-first-order kinetics of COD removal. **(C)** NO_3_^−^, SO_4_^2−^, PO_4_^3−^, and Cl^−^ during electrolysis of domestic wastewater at a current density of 14 mA cm^−2^ and recirculation flow rate of 1.5 L min^−1^.

Figure 5A shows the mineralization of organic matter present in domestic wastewater samples studied by analyzing total organic carbon (TOC) during 7 h of electro-oxidation under optimal operating conditions (Q = 1.5 L min^−1^ and j = 14 mA cm^−2^), achieving 44.9% of mineralization. The concentration of TOC was reduced from 103 mg L^−1^ TOC to 57 mg L^−1^ TOC. Although some intermediates could be formed during the degradation process, these intermediates were not identified in this work due to the complexity of the sample. In addition, the degradation kinetic constant indicates that the mineralization follows pseudo-first-order kinetics (*k* = 0.0076 min^−1^) with R^2^ = 0.9854, as shown in the inset of Figure 5A. Finally, the results showed an effective degradation efficiency, which means that the majority of organic matter was transformed to CO_2_, H_2_O, and ionic compounds. Therefore, the use of a DiaClean^®^ cell was highly effective for the removal of organic matter in wastewater samples. The total mineralization of organic compounds present in domestic wastewater is very difficult to achieve in short electrolysis times since, in addition to detergents, there is a complex variation of persistent organic compounds such as drugs, pesticides, and insecticides. However, electrochemical processes have the advantage of oxidizing organic matter directly and indirectly. Direct oxidation occurs by the direct transfer of electrons from organic matter to the surface of the electrode, and indirect oxidation occurs through the generation of HORS that are formed on the electrode surface during electrolysis, e.g.,^•^OH, ${{CO}_{3}^{•-},{C_{2}O}_{6}^{2-},S_{2}O_{8}^{2-},SO}_{4}^{•-}$ , Cl_2_, HClO, ClO^−^, ${Cl}^{•}$ , $ClO_{2}^{•}$ $O_{2}^{•-}$ , ^1^O_2_, ClO_2_, and ${P_{2}O}_{8}^{4-}$ shown in reactions 2, 4, 7, 9, and 10–19, respectively (Cañizares et al., 2005; Sánchez et al., 2013; Ruiz et al., 2014; Jasper et al., 2017; Ganiyu & Martínez-Huitle, 2019; Ganiyu & El-din, 2020).

In addition, Table 1 summarizes the final characterization of domestic wastewater analyzed using the optimal operating parameters of 1.5 L min^–1^ as feed flow and 50 mA cm^–2^. The results showed that all parameters comply with current environmental regulations regarding discharges to a freshwater body. Therefore, it is concluded that this treatment can be applied to treat domestic wastewater after the conventional process to be able to remove persistent organic pollutants such as detergents as very satisfactory results were obtained. In turn, the treatment reduces the concentration of COD, TOC, and turbidity present in wastewater, and the control parameters (pH, temperature, and conductivity) are kept within permissible limits.

**TABLE 1:** Physicochemical characterization of domestic wastewater before and after electrochemical treatment.

	COD (mg L^−1^)	Surfactant (mg L^−1^)	Chloride (mg L^−1^)	Sulfate (mg L^−1^)	Phosphate (mg L^−1^)	Nitrate (mg L^−1^)	TOC (mg L^−1^)	Temp. (°C)	pH	Conductivity (μs cm^−1^)	Turbidity (NTU)
C_O_	102.54	12.35	54.71	38.53	3.46	5.10	103	13.2	8.09	736.33	135
C_f_	56.53	4.36	7.59	53.67	3.16	7.87	57	24.8	9.38	646.67	101
MPV*	250	0.5					—	<35	5-9	—	—

* Maximum permitted value: Ecuadorian environmental legislation.

### 3.4 Ecotoxicity test with microalgae Chlorella sp.

**FIGURE 6:**
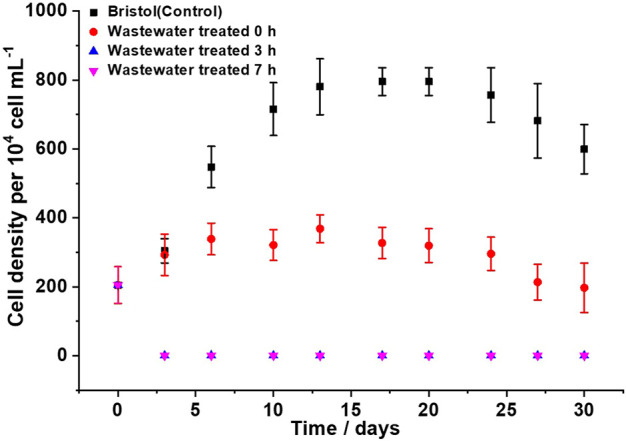
Growth curves of *Chlorella sp*. culture in Bristol medium, domestic wastewater, and domestic wastewater treated by electro-oxidation for 3 and 7 h.

*Chlorella* microalgae have been extensively studied due to their ability to integrate contaminants into their metabolism. Despite the large amount of nutrients such as phosphorus, nitrogen, and heavy metals contained in wastewater, *Chlorella* can grow in the culture medium and absorb these compounds. It is evident from Figure 6 that *Chlorella* early adapts to wastewater (without treatment) and shows exponential growth during the first 6 days of treatment, reaching a maximum cell density of 47% smaller than that of the control medium (Bristol). Velegraki et al. (2010) showed that the untreated solution was partially toxic for the studied microorganism, and during the first stages of electrochemical oxidation, the toxicity increased, inhibiting the test microorganism by 80% after 30 min of exposure. Figure 5 shows that *Chlorella* could not grow in AEO-treated wastewater (neither 3- nor 7-h electrolysis). By the third day of cell counting, a cell density of 0 × 10^4^ cells ml^−1^ was found in both treatment processes involving treated wastewater. Sometimes, by-products generated during degradation can be more toxic than the original contaminant (Velegraki et al., 2010). Mora Gómez (2020) compared ceramic and BDD electrodes and showed that BDD electrode-treated solutions produce more toxic by-products. On the other hand, it has been reported that during electrolysis with BDD, HORS (e.g., ^•^OH, ${{CO}_{3}^{•-},{C_{2}O}_{6}^{2-},S_{2}O_{8}^{2-},SO}_{4}^{•-}$ , Cl_2_, HClO, ClO^−^, ${Cl}^{•}$ , $ClO_{2}^{•}$ $O_{2}^{•-}$ , ^1^O_2_, ClO_2_, and ${P_{2}O}_{8}^{4-}$ shown in reactions 2, 4, 7, 9, and 10–19, respectively) can be generated which facilitate degradation (Cañizares et al., 2005; Sánchez et al., 2013; Ruiz et al., 2014; Jasper et al., 2017; Ganiyu & Martínez-Huitle, 2019; Ganiyu & El-din, 2020) but at the same time generate a toxic environment for the growth of the microalgae such as *Chlorella*. In this work, the presence of ${NO}_{3}^{-},{SO}_{4}^{2-}$ , ${PO}_{4}^{3-}$ y Cl^−^ was evaluated in the initial sample, during and at the end of electrolysis (Table 1; Figure 5C). It is observed that during electrolysis, Cl^−^ decreases, ${SO}_{4}^{2-}$ increases slightly, ${PO}_{4}^{3-}$ decreases slightly, and ${NO}_{3}^{-}$ oscillates (Figure 5C). The decrease in Cl^−^ during electrolysis could be due to the formation of Cl_2_(gas) and organochlorine molecules; ${SO}_{4}^{2-}$ increases because surfactants release this ion during oxidation, while the concentration of ${PO}_{4}^{3-}$ practically remains constant, since pH 9.8 (reaction 19) is not favored (it is favored in very alkaline medium) (Cañizares et al., 2005; Sánchez et al., 2013).

### 3.5 Analysis of operational conditions and operational cost

Table 2 shows the energy consumption and operating costs in the removal of surfactants by different technologies (Collivignarelli et al., 2019; Khosravanipour Mostafazadeh et al., 2019; Kong et al., 2006; Patil et al., 2020). In this work, the operating cost was calculated under optimal conditions for the DiaClean^®^. The energy consumption of the DiaClean^®^ cell and the energy consumption of the peristaltic pump were taken into account. The energy in kilowatt-hours was obtained by multiplying power in kW and the time of electrolysis in hours. Finally, the cost of operation was calculated, citing a cost of 0.063 USD per kilowatt-hour (average in Ecuador, January–December of 2018–2017). The results showed that the energy used during the electrolysis under the optimal conditions (1.5 L min^−1^ and 140 mA cm^−2^) was 2.59 kW h m^−3^, and the operation cost was $1.40 USD m^−3^ (Table 2), which means that the use of the DiaClean^®^ cell could be connected to sustainable energy sources due to its low energy consumption. AEO is not the cheapest technology for the treatment of domestic wastewater with a high detergent load (Table 2). However, not being the most expensive technology, it could run on solar energy, making it cheaper and more environmentally friendly.

**TABLE 2:** Energy consumption and operating costs involved in the removal of surfactants by different technologies.

Treatment technique	Energy consumption (kW h m^−3^)	Cost (USD m^−3^)	Bibliography
Hydrodynamic cavitation	30	3.3	Patil et al. (2020)
Electro-oxidation of ultrafiltration concentrate	4–22	0.90	Khosravanipour Mostafazadeh et al. (2019)
Electrochemical oxidation of modified Ti/Co/SnO_2_–Sb_2_O_3_ + kaolin particles	700	-	Kong et al. (2006)
TAMR-NF*	10–45.5	1–6	Collivignarelli et al. (2019)
AEO	2.59	1.40	This study

*Thermophilic aerobic membrane reactor (TAMR), nanofiltration (NF).

## 4 Conclusion

Advanced electrochemical oxidation, using a DiaClean^®^ cell equipped with a BDD anode, allows for the remediation of domestic wastewater with a high load of surfactants. The best operating conditions of the DiaClean^®^ cell coupled to a recirculation system were: flow rate = 1.5 L min^−1^ and current density j = 14 mA cm^−2^. Under these conditions, a removal of 44.9% of TOC, 64.7% of COD, and 48.7% of surfactant degradation was achieved after 7 h of electrolysis. In domestic wastewater treatment with a high detergent load, it is key to control the applied temperature and current density to avoid excessive foaming. The microalgae toxicity study showed that municipal wastewater treated by AEO can be toxic to biota due to the formation of highly reactive species during electrolysis, such as ${{CO}_{3}^{•-},{C_{2}O}_{6}^{2-},S_{2}O_{8}^{2-},SO}_{4}^{•-}$ , Cl_2_, HClO, and ${P_{2}O}_{8}^{4-}$ . The energy consumption of the DiaClean^®^ cell is less than that of the peristaltic pump in the recirculation system, and the operating cost of the system under the optimal operating conditions was $1.40 USD m^−3^. Based on the results obtained, advanced electro-oxidation is presented as a very promising technology in the near future. In addition, its feasibility and availability make the electrochemical process an excellent alternative for the remediation of wastewater, which is linked to the principles of sustainability and preservation of water.

## Data Availability

All data generated in this research has been included in the article, there is no additional data elsewhere.
